# Is hypoxemia explained by intracardiac or intrapulmonary shunt in COVID-19-related acute respiratory distress syndrome?

**DOI:** 10.1186/s13613-020-00726-z

**Published:** 2020-08-06

**Authors:** Paul Masi, François Bagate, Thomas d’Humières, Lara Al-Assaad, Laure Abou Chakra, Genevieve Derumeaux, Armand Mekontso Dessap

**Affiliations:** 1 Service de Médecine Intensive Réanimation, Assistance Publique-Hôpitaux de Paris, Hôpitaux Universitaires Henri Mondor, 51, avenue du Maréchal de Lattre de Tassigny, 94010 Créteil Cedex, France; 2 Groupe de recherche clinique CARMAS, Faculté de Santé, Université Paris Est Créteil, 94010 Créteil, France; 3 Service de Physiologie, AP-HP, Hôpitaux Universitaires Henri Mondor, 94010 Créteil, France; 4 INSERM U955, Université Paris-Est, Créteil, France

## Abstract

Hypoxemia is the main feature of COVID-19-related acute respiratory distress syndrome (C-ARDS), but its underlying mechanisms are debated, especially in patients with low respiratory system elastance (Ers). We assessed 60 critically ill patients hospitalized in our intensive care unit for C-ARDS. We used contrast transthoracic echocardiography to assess patent foramen ovale (PFO) shunt and transpulmonary bubble transit (TPBT). The median Ers was 32 cmH_2_O/L. PFO shunt was detected in six (10%) patients and TPBT in 12 (20%) patients. PFO shunt and TPBT were similar in patients with higher or lower Ers. In conclusion, PFO and TPBT do not seem to be the main drivers of hypoxemia in C-ARDS, especially in patients with lower Ers.

Dear Editor

Hypoxemia is the main feature of COVID-19-related acute respiratory distress syndrome (C-ARDS). Characteristically, some patients with C-ARDS maintain relatively preserved respiratory mechanics despite severe hypoxemia [1]. Two distinct pathophysiology phenotypes have been suggested: type H (with high elastance, high lung weight, and high lung recruitability), and type L (with low elastance, low lung weight, and low lung recruitability) [1]. Although patients with low or high respiratory elastance may both experience severe hypoxemia, the underlying mechanisms of hypoxemia may differ between groups [1].

Shunt across patent foramen ovale (PFO) is reported in ARDS cases, often associated with poor response to positive expiratory pressure [2]. In ARDS, intrapulmonary shunt, seen as transpulmonary bubble transit (TPBT), may occur in perfused non-aerated lung areas secondary to pulmonary vessels dilatation [3]. The aim of the present study was to assess the role of intracardiac or intrapulmonary shunt in C-ARDS-related hypoxemia, and their possible association with C-ARDS respiratory mechanics.

We studied patients who had Berlin-defined ARDS [4] and laboratory-confirmed Covid-19 infection, and who were admitted to our ICU between March 8th and April 14th, 2020. Transthoracic echocardiography was performed on all patients by trained operators (competent in advanced critical care echocardiography) within one [1–3] days of ICU admission. The right-to-left shunt was evaluated on the four-chamber view by injecting 9.5 mL of sterile modified fluid gelatine solution (Plasmion, Fresenius-Kabi, Sevres, France) aerated with 0.5 mL of room air via two syringes connected to a three-way stopcock [2]. The injection was considered successful if the entire right atrium was opacified with microbubbles-induced contrast. Up to three successful contrast studies were performed on each patient. The majority of patients (51/60) had their right atrium opacified via superior vena cava-threaded catheter. Moderate-to-large PFO shunt was defined as right-to-left passage of at least ten bubbles through the valve-like structure within three cardiac cycles after complete opacification of the right atrium [2]. Moderate-to-large TPBT was defined as right-to-left passage of at least ten bubbles through a pulmonary vein more than three cardiac cycles after complete opacification of the right atrium [3]. Given that we routinely use echocardiography to assess the circulatory status of mechanically ventilated ARDS patients in our ICU, this study was considered as a component of standard care and patient’s consent was waived.

We investigated 60 critically ill patients hospitalized in our ICU for ARDS with confirmed SARS-CoV-2 infection. PFO shunt was detected in six patients (prevalence 10%, 95% confidence interval (CI) 2–17%). TPBT was detected in 12 patients (prevalence 20%, 95% CI 9–30%). Demographic characteristics, respiratory mechanics, and hemodynamic parameters of patients are presented in Table 1, according to whether their respiratory system elastance (Ers) was lower or higher than the median value of 32 cmH_2_O/L. PFO shunt and TPBT were similar in both groups (Table 1, Additional file 1: Figure S1). Driving pressure was higher, whereas PaO_2_/FiO_2_ ratio was lower in the latter group (Table 1).

**Table 1:** Demographic characteristics, respiratory mechanics, and hemodynamic parameters, according to respiratory system elastance

Characteristic	All patients *n* = 60	Lower Ers *n* = 30	Higher Ers *n* = 30	*P* value
Age (y)	62 [51–72]	64 [50–73]	58 [53–69]	0.36
Male gender (%)	50 (83)	27 (90)	23 (77)	0.17
Body mass index (kg/m^2^)	27 [24–32]	28 [24–32]	27 [25–31]	0.76
PEEP (cmH_2_0)	11 [9–12]	12 [9–12]	10 [8–12]	0.13
Driving pressure (cmH_2_O)	12 [11–15]	11 [9–12]	14 [13–16]	< 0.001
Crs (mL/cmH_2_O)	32 [27–40]	40 [36–45]	27 [21–30]	< 0.001
Ers (cmH_2_O/L)	32 [24–34]	25 [22–28]	38 [33–48]	< 0.001
PaO_2_/FiO_2_ (mmHg)	142 [107–194]	170 [131–224]	119 [72–177]	< 0.01
PaCO_2_ (mmHg)	42 [38–47]	42 [36–46]	44 [40–51]	0.07
LVEF (%)	60 [49–6]	60 [49–68]	60 [48–70]	0.88
Cardiac index (L/min/m^2^)	2.78 [2.42–3.40]	2.69 [2.39–3.23]	2.95 [2.44–3.71]	0.24
RV/LV area ratio	0.6 [0.50–0.70]	0.6 [0.50–0.73]	0.58 [0.49–0.70]	0.96
Acute cor pulmonale (%)	26 (43)	13 (43)	13 (43)	> 0.99
PFO shunt (%)	6 (10)	2 (7)	4 (13)	0.67
TPBT (%)	12 (20)	8 (27)	4 (13)	0.20

Ers: respiratory system elastance; PEEP: positive end-expiratory pressure; Crs: respiratory system compliance; PaO_2_/FiO_2_: ratio of partial pressure of oxygen in arterial blood and fraction of inspired oxygen; PaCO_2_: partial pressure of carbon dioxide in arterial blood; LVEF: left ventricle ejection fraction; RV: right ventricle; LV: left ventricle; PFO: patent foramen ovale; TPBT: transpulmonary bubble transit

Mann–Whitney test for continuous variables and Chi-square test or Fisher exact test for categorical variables

Our prevalence of PFO shunt in C-ARDS (10%) is close to that described in other types of ARDS upon using transthoracic echocardiography (TTE) (7%), but lower than that detected upon using trans-oesophageal echocardiography (TEE) (16–19%) [2, 3]. This discrepancy between TTE and TEE has previously been described [5]. The lack of sensitivity of TTE is probably related to the effect of numerous interference sources on image acquisition from the chest wall of ventilated ARDS patients. PFO shunt does not seem to play a stronger role in inducing hypoxemia in C-ARDS than in other forms of ARDS.

Concerning TPBT prevalence in C-ARDS, we found 20%, which is similar to that reported in other forms of ARDS (26%) [3]. The mechanisms by which TPBT occurs include transit through perfused, non-aerated lung areas, transit through dilated capillaries, or via dynamic intrapulmonary arteriovenous anastomosis channels. SARS-COV-2 virus enters host cells via angiotensin converting enzyme 2 receptors, which are widely expressed on endothelial cells including those present in the lung vasculature. As a result, SARS-CoV-2 infection may induce endotheliitis altering pulmonary vascular homeostasis [6]. Not having massive TPBT in C-ARDS patients does not formally exclude dysregulation of hypoxic vasoconstriction and/or mild dilatation of pulmonary vessels secondary to SARS-CoV-2 infection. However, the diameter of microbubbles resulting from agitated colloids, used for TTE, is smaller than that obtained with agitated saline, which may account for a higher perceived TPBT.

Our study has several limitations. First, screening for PFO shunt and TPBT by TTE (and not by TEE) decreased the sensitivity of detection [5]. The pandemic nature of C-ARDS thwarted the application of TEE in routine care, and our results need to be confirmed by such a sensitive tool to better scrutinize the exact pathophysiology of vascular shunt. TPBT does not precisely depict the anatomical sites of intrapulmonary shunt. Moreover, the huge workload we had during the pandemic disabled us from repeating the micro-bubble studies over time as PEEP changed or disease progressed. Besides, our definition of PFO shunt and TPBT based on the number of bubbles to evaluate hypoxemia may be questionable, and we could not assess the inter-observer reproducibility of PFO shunt and TPBT using TTE. Second, our sample size was limited, however our report represents the largest echocardiography-based study on C-ARDS patients to date. Third, we only used raw surrogates (respiratory system elastance) to assess the C-ARDS phenotype. Further studies with enhanced phenotyping are needed to evaluate the possible association between PFO shunt or TPBT and the specific C-ARDS phenotypes.

In conclusion, PFO shunt and TPBT do not seem to be the main drivers of hypoxemia in C-ARDS, especially in patients with lower Ers.

## Supplementary information

**Additional file 1: Figure S1.** Prevalence of patent foramen ovale (PFO) shunt and transpulmonary bubble transit (TPBT) in patients with COVID-19 acute respiratory distress syndrome according to respiratory system elastance (Ers).

## Data Availability

All data generated and analysed during the study are included in the published article and can be shared upon request. All authors helped to revise the draft of the manuscript.
