# Supplementary material for: Is hypoxemia explained by intracardiac or intrapulmonary shunt in COVID-19-related acute respiratory distress syndrome?
Source: Ann Intensive Care. 2020 Aug 6;10:108. doi: 10.1186/s13613-020-00726-z (PMC7407421; doi:10.1186/s13613-020-00726-z)
Supplement: Supplementary file 1 — Additional file 1: Figure S1. Prevalence of patent foramen ovale (PFO) shunt and transpulmonary bubble transit (TPBT) in patients with COVID-19 acute respiratory distress syndrome according to respiratory system elastance (Ers). [file 13613_2020_726_MOESM1_ESM.docx]

Figure S1. Prevalence of patent foramen ovale (PFO) shunt and transpulmonary bubble transit

(TPBT) in patients with COVID-19 acute respiratory distress syndrome according to

respiratory system elastance (Ers).
